# Making sense of vacancy correlations with single-crystal diffuse scattering data

**DOI:** 10.1107/S2052252520008544

**Published:** 2020-06-30

**Authors:** Matthew Krogstad

**Affiliations:** a Argonne National Laboratory, Materials Science Division, 9700 S. Cass Avenue, Lemont, IL 60439, USA

**Keywords:** defective half-Heuslers, diffuse scattering, short-range order, correlated disorder

## Abstract

The results of Roth *et al.* [*IUCrJ* (2020). **7**, 673–680] provide a clear picture of occupational correlations in half-Heusler compounds.

Local ordering of atoms within a crystalline structure is increasingly recognized as essential to understanding the properties of a variety of interesting materials. This is shown by the success of pair distribution function (PDF) analysis of scattering data from powder samples to investigate departures from perfectly crystalline order (Billinge & Kanatzidis, 2004 ▸; Egami & Billinge, 2012 ▸). Diffuse scattering from single crystals can also be a powerful probe of local order, potentially providing key information that would be difficult to obtain from PDF methods; however, difficulties in both measurement and interpretation of data have too often restricted their use by a larger community. In this issue of **IUCrJ**, Roth *et al.* (2020 ▸) demonstrate how simple concepts can be applied to better understand single-crystal diffuse scattering from half-Heusler systems.

Recent developments in instrumentation and computing have made the measurement of single-crystal diffuse scattering much easier than it was even ten years ago (Ye *et al.*, 2018 ▸; Keen *et al.*, 2015 ▸). Valuable data can also be obtained with lab-based X-ray sources and electron sources, which are able to produce high-quality data in a carefully chosen set of planes (Izquierdo *et al.*, 2011 ▸; Welberry & Goossens, 2016 ▸). However, the analysis of such data can still seem a daunting task, especially when compared to the powder-based PDF data. A key step for proposed solutions is comparison of experimental scattering data with those from simulated structures, but it can be difficult to connect diffuse scattering from simulated structures to specific structural motifs.

Roth *et al.* (2020 ▸) have produced a compelling analysis of diffuse scattering from half-Heusler materials Nb_1 − *x*_CoSb. These have proven challenging to understand via crystallographic techniques; while crystallographic solutions can confirm the concentration of vacancies on the Nb sites, the distribution of these vacancies is more difficult to discern. Electron diffraction data collected by Xia *et al.* (2018 ▸, 2019 ▸) show clear diffuse scattering patterns over a range of concentrations, implying that vacancies are spatially correlated locally without being ordered over the entire structure. Simulations based on density functional theory (DFT) have reproduced the scattering for certain concentrations but cannot be generalized over the full range of vacancy concentrations and provide limited insight into the systematics of local structures in these systems.

Roth *et al.* (2020 ▸) approach these data starting with the simple concept that Nb site vacancies will tend to avoid one another. Simulated crystals are then generated by a Monte Carlo procedure which allows vacancies to move and penalizes vacancies being nearest-neighbors and next-nearest neighbors via a simple Ising-like model. Diffuse scattering from these models is rapidly calculated using the program *SCATTY* (Paddison, 2019 ▸). While the number of individual states produced by these models is quite large, the only free parameters are the vacancy concentration, the ratio between energies associated with nearest and next-nearest neighbors, and the temperature.

For a single ratio of energies, these models impressively reproduce published electron diffraction data over a range of vacancy concentrations (see Fig. 1 ▸). Distinct ordered ground states are reproduced at *x* = 1/4 and *x* = 1/6, with intermediate compositions showing short-range combinations of the two. For concentrations with *x* < 1/6, broader wave-like scattering is seen. This entire range of compositions can be modeled and explained with self-avoiding vacancies.

These results provide a significant advance in the knowledge of the structure of these materials as well as a great example of how short-range ordering can be understood, modeled and described. Roth *et al.* (2020 ▸) have provided a clear picture of occupational correlations in half-Heusler compounds. With single-crystal diffuse scattering data becoming easier to obtain, the use of simple models of short-range substitutional correlations is a key method for understanding such data.

## Figures and Tables

**Figure 1:**
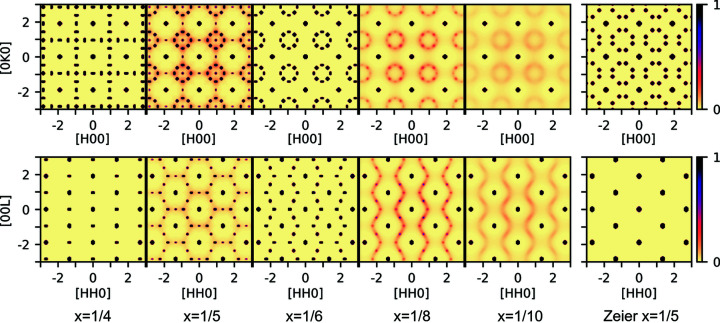
Scattering data from simulated crystals of Nb_1 − *x*_CoSb from Roth *et al.* (2020 ▸). For *x* = 1/4, an ordered state is produced where there are no nearest-neighbor vacancies, producing a commensurate set of superstructure peaks, and for *x* = 1/6, next-nearest neighbors can be completely avoided as well, producing eight-point rings when appropriately twinned. For concentrations with 1/4 > *x* > 1/6, a disordered mixture of the two states is shown to minimize energy compared to proposed ordered states and reproduces observed scattering in this range. For concentrations with *x* < 1/6, disordered states produce broad rings and waves.
